# Supplementary material for: Crystal structure of the 4-hydroxybutyryl-CoA synthetase (ADP-forming) from nitrosopumilus maritimus
Source: Commun Biol. 2024 Oct 21;7:1364. doi: 10.1038/s42003-024-06432-x (PMC11494057; doi:10.1038/s42003-024-06432-x)
Supplement: Supplementary file 3 — Description of Additional Supplementary Files [file 42003_2024_6432_MOESM3_ESM.pdf]

# Description of Additional Supplementary Files

**File name:** Supplementary Data 1
**Description:** Full list of sequences used for phylogenetic tree generation.

**File name:** Supplementary Data 2
**Description:** Fasta file for Codon Optimized 0206 gene.
