# Supplementary material for: Crystal structure of the 4-hydroxybutyryl-CoA synthetase (ADP-forming) from nitrosopumilus maritimus
Source: Commun Biol. 2024 Oct 21;7:1364. doi: 10.1038/s42003-024-06432-x (PMC11494057; doi:10.1038/s42003-024-06432-x)
Supplement: Supplementary file 4 — Supplementary Data 1 [file 42003_2024_6432_MOESM4_ESM.pdf]

Supplementary Table 1: Sequence of Nmar_0206 homologues.

Organism Name	Protein name	Accession Number	Domain/Phylum/Class
Aciduliprofundum boonei	acetyl coenzyme A synthetase (ADP forming)	WP_008084432.1	Archaea/Euryarchaeota/DHVE2
Bellilinea caldifistulae	acetyl-CoA synthetase	WP_061917260.1	Bacteria/Chloroflexota/Anaerolineae
Brevefilum fermentans	acetate–CoA ligase family protein	WP_197687098.1	Bacteria/Chloroflexota/Anaerolineae
Candidatus Eisenbacteria	acetate–CoA ligase family protein	WP_008084432.1	Bacteria/Chloroflexota/Anaerolineae
Chloroflexi bacterium	acetate–CoA ligase family protein	NLG99206.1	Bacteria/Chloroflexota
Leptolinea sp.	acetate–CoA ligase family protein	NMB56267.1	Bacteria/Chloroflexota/Anaerolineae
Leptolinea tardivitalis	acetate–CoA ligase family protein	WP_062421259.1	Bacteria/Chloroflexota/Anaerolineae
Levilinea saccharolytica	acetate–CoA ligase family protein	WP_062418633.1	Bacteria/Chloroflexota/Anaerolineae
Candidatus Methanomassiliicoccus intestinalis	acetyltransferase	WP_020448504.1	Archaea/Euryarchaeota/Thermoplasmata
Haloferax volcanii	acetyl-CoA synthetase	WP_004043943.1	Archaea/Euryarchaeota/Halobacteria
Methanobacterium congolense	acetate–CoA ligase family protein	WP_071906649.1	Archaea/Euryarchaeota/Methanobacteria
Methanobacterium formicicum	acetate–CoA ligase family protein	WP_004030442.1	Archaea/Euryarchaeota/Methanobacteria
Methanobacterium petrolearium	acetate–CoA ligase family protein	WP_209625374.1	Archaea/Euryarchaeota/Methanobacteria
Methanobacterium sp.	CoA-binding domain-containing protein	WP_292756096.1	Archaea/Euryarchaeota/Methanobacteria
Methanobacterium subterraneum	acetate–CoA ligase family protein	WP_169032909.1	Archaea/Euryarchaeota/Methanobacteria
Methanocella arvoryzae	acetate-CoA ligase	WP_ 012036318.1	Archaea/Euryarchaeota/Methanomicrobia

Methanocella conradii	acetate-CoA ligase	WP_014404925.1	Archaea/Euryarchaeota/Methanomicrobia
Methanocella paludicola	acetate–CoA ligase family protein	WP_012901660.1	Archaea/Euryarchaeota/Methanomicrobia
Methanococcoides burtonii	acetate-CoA ligase	WP_011499410.1	Archaea/Euryarchaeota/Methanomicrobia
Methanococcoides methylutens	acetate-CoA ligase	WP_048205925.1	Archaea/Euryarchaeota/Methanomicrobia
Methanococcoides orientis	acetate–CoA ligase family protein	WP_233084021.1	Archaea/Euryarchaeota/Methanomicrobia
Methanococcoides seepicolus	acetate–CoA ligase family protein	WP_250868540.1	Archaea/Euryarchaeota/Methanomicrobia
Methanohalobium evestigatum	acetyl coenzyme A synthetase	WP_013195317.1	Archaea/Euryarchaeota/Methanomicrobia
Methanohalophilus euhalobius	acetate-CoA ligase	WP_096712683.1	Archaea/Euryarchaeota/Methanomicrobia
Methanohalophilus levihalophilus	acetate–CoA ligase family protein	WP_209682318.1	Archaea/Euryarchaeota/Methanomicrobia
Methanohalophilus mahii	acetate-CoA ligase	WP_013038092.1	Archaea/Euryarchaeota/Methanomicrobia
Methanohalophilus portucalensis	acetate-CoA ligase	WP_072360490.1	Archaea/Euryarchaeota/Methanomicrobia
Methanohalophilus profundi	acetate-CoA ligase	WP_129598452.1	Archaea/Euryarchaeota/Methanomicrobia
Methanolobus bombayensis	acetate–CoA ligase family protein	WP_209618454.1	Archaea/Euryarchaeota/Methanomicrobia
Methanolobus chelungpuianus	acetate–CoA ligase family protein	WP_256621754.1	Archaea/Euryarchaeota/Methanomicrobia
Methanolobus halotolerans	acetate-CoA ligase	WP_135389970.1	Archaea/Euryarchaeota/Methanomicrobia
Methanolobus profundi	acetate-CoA ligase	WP_091937667.1	Archaea/Euryarchaeota/Methanomicrobia
Methanolobus psychrophilus	acetyl coenzyme A synthetase	AFV23390.1	Archaea/Euryarchaeota/Methanomicrobia
Methanolobus psychrotolerans	acetate-CoA ligase	WP_094228889.1	Archaea/Euryarchaeota/Methanomicrobia
Methanolobus tindarius	acetate-CoA ligase	WP_023845745.1	Archaea/Euryarchaeota/Metha

			nomicrobia
Methanolobus zinderi	acetate-CoA ligase	WP 176964306.1	Archaea/Euryarchaeota/Methanomicrobia
Methanomethylovorans hollandica	acetate–CoA ligase family protein	WP_015324018.1	Archaea/Euryarchaeota/Methanomicrobia
Methanosalsum natronophilum	acetate–CoA ligase family protein	WP_259134952.1	Archaea/Euryarchaeota/Methanomicrobia
Methanosalsum zhilinae	acetate-CoA ligase	WP_013898132.1	Archaea/Euryarchaeota/Methanomicrobia
Armatimonadetes bacterium	acetate–CoA ligase family protein	MBC8157135.1	Bacteria/Armatimonadota/undefined
Candidatus Nitrosarchaeum limnium	acetate–CoA ligase family protein	WP_010190675.1	Archaea/Nitrososphaerota/Nitrososphaeria
Candidatus Nitrosopelagicus brevis	CoA-binding protein	NMI83880.1	Archaea/Nitrososphaerota/Nitrososphaeria
Candidatus Nitrosopelagicus sp.	acylCoA synthetase	GIT55543.1	Archaea/Nitrososphaerota/Nitrososphaeria
Candidatus Nitrosopolaris wilkensis	acylCoA synthetase	PWU81816.1	Archaea/Nitrososphaerota/Nitrososphaeria
Candidatus Nitrosopumilus koreensis	3-hydroxypropionyl-CoA synthetase (ADP-forming)	WP_007551149.1	Archaea/Nitrososphaerota/Nitrososphaeria
Candidatus Nitrosopumilus sediminis	acetate-CoA ligase	WP_014964350.1	Archaea/Nitrososphaerota/Nitrososphaeria
Candidatus Nitrososphaera evergladensis	4-hydroxybutyryl-CoA synthetase (ADP-forming)	WP_148699949.1	Archaea/Nitrososphaerota/Nitrososphaeria
Candidatus Nitrososphaera gargensis	acetate–CoA ligase family protein	WP_015020118.1	Archaea/Nitrososphaerota/Nitrososphaeria
Candidatus Nitrosotalea devanaterra	4-hydroxybutyryl-CoA synthetase (ADP-forming)	CUR50945.1	Archaea/Nitrososphaerota/Nitrososphaeria
Candidatus Nitrosotalea bavarica	acetate–CoA ligase family protein	WP_101477917.1	Archaea/Nitrososphaerota/Nitrososphaeria
Candidatus Nitrosotalea devanaterra	4-hydroxybutyryl-CoA synthetase (ADP-forming)	CUR50945.1	Archaea/Nitrososphaerota/Nitrososphaeria

Candidatus Nitrosotalea okcheonensis	acetate–CoA ligase family protein	WP_157928055.1	Archaea/Nitrososphaerota/Nitrososphaeria
Candidatus Nitrosotalea sinensis	acetate–CoA ligase family protein	WP_101008906.1	Archaea/Nitrososphaerota/Nitrososphaeria
Candidatus Nitrosotenuis aquarius	acetate-CoA ligase	WP_100182658.1	Archaea/Nitrososphaerota/Nitrososphaeria
Candidatus Nitrosotenuis chungbukensis	acetate–CoA ligase family protein	WP_042683670.1	Archaea/Nitrososphaerota/Nitrososphaeria
Candidatus Nitrosotenuis cloacae	acetate–CoA ligase	WP_048186944.1	Archaea/Nitrososphaerota/Nitrososphaeria
Candidatus Nitrosotenuis sp.	CoA-binding protein	TBR22739.1	Archaea/Nitrososphaerota/Nitrososphaeria
Candidatus Nitrosotenuis uzonensis	acetate–CoA ligase family protein	WP_048194424.1	Archaea/Nitrososphaerota/Nitrososphaeria
Cenarchaeum symbiosum	acyl-CoA synthetase (NDP- forming)	ABK76672.1	Archaea/Nitrososphaerota/Nitrososphaeria
Nitrosarchaeum koreense	acetate–CoA ligase family protein	WP_007549553.1	Archaea/Nitrososphaerota/Nitrososphaeria
Nitrosopumilus cobalaminigenes	acetate–CoA ligase	WP_179361083.1	Archaea/Nitrososphaerota/Nitrososphaeria
Nitrosopumilus maritimus (strain SCM1)	3-hydroxypropionyl-CoA synthetase (ADP-forming)	WP_012215692.1	Archaea/Nitrososphaerota/Nitrososphaeria
Nitrosopumilus maritimus (strain SCM1)	4-hydroxybutyryl-CoA synthetase (ADP-forming)	WP_012214589.1	Archaea/Nitrososphaerota/Nitrososphaeria
Nitrosopumilus oxyclinae	acetate–CoA ligase family protein	WP_179362924.1	Archaea/Nitrososphaerota/Nitrososphaeria
Nitrosopumilus salaria	4-hydroxybutyryl-CoA synthetase (ADP-forming)	WP_008297726.1	Archaea/Nitrososphaerota/Nitrososphaeria
Nitrosopumilus sp.	4-hydroxybutyryl-CoA synthetase (ADP-forming)	RMW38333.1	Archaea/Nitrososphaerota/Nitrososphaeria
Nitrososphaera viennensis	4-hydroxybutyryl-CoA synthetase (ADP-forming)	WP_075055435.1	Archaea/Nitrososphaerota/Nitrososphaeria
Candidatus Nitrosotalea okcheonensis	acetate–CoA ligase family protein	WP_157928055.1	Archaea/Nitrososphaerota/Nitrososphaeria

Candidatus Nitrosocosmicus oleophilus	acetate–CoA ligase family protein	WP_196817532.1	Archaea/Nitrososphaerota/Nitrososphaeria
Staphylothermus hellenicus	acetyl coenzyme A synthetase (ADP forming)	ADI31488.1	Archaea/Thermoproteota/Thermoprotei
Ignisphaera aggregans	acetyl coenzyme A synthetase (ADP forming)	ADM28387.1	Archaea/Thermoproteota/Thermoprotei
Pyrolobus fumarii	CoA-binding domain protein	AEM39269.1	Archaea/Thermoproteota/Thermoprotei
Thermogladius calderae	CoA-binding domain protein	AFK51136.1	Archaea/Thermoproteota/Thermoprotei
Desulfurococcales archaeon	CoA-binding protein	MCD6324124.1	Archaea/Thermoproteota/Thermoprotei
Candidatus Bathyarchaeota archaeon	acetyl coenzyme A synthetase (ADP forming)	HDM23685.1	Archaea/Bathyarchaeota/undefined
Acidilobales archaeon	acetyl coenzyme A synthetase (ADP forming)	HDN75497.1	Archaea/Thermoproteota/Thermoprotei
Fervidicoccaceae archaeon	CoA-binding protein	MCC6010103.1	Archaea/Thermoproteota/Thermoprotei
Staphylothermus sp.	CoA-binding protein	MCD6196563.1	Archaea/Thermoproteota/Thermoprotei
Desulfurococcales archaeon	CoA-binding protein	MCD6488109.1	Archaea/Thermoproteota/Thermoprotei
Thermoprotei archaeon	CoA-binding protein	MCI4396430.1	Archaea/Thermoproteota/Thermoprotei
Ignisphaera sp.	CoA-binding protein	MCI4436598.1	Archaea/Thermoproteota/Thermoprotei
Crenarchaeota archaeon	acetyl-CoA synthetase (ADP forming)	NPA97047.1	Archaea/Thermoproteota/Thermoprotei
Aigarchaeota archaeon NZ13 MG1	acetyl-CoA synthetase (ADP forming)	PUA31671.1	Archaea/Aigarchaeota/undefined
Zestosphaera tikiterensis	acetyl-CoA synthetase (ADP forming)	PUA33430.1	Archaea/Thermoproteota/Thermoprotei
Deltaproteobacteria bacterium	acetyl-CoA synthetase (ADP forming)	RLB79341.1	Archaea/Deltaproteobacteria/undefined

Candidatus Verstraetearchaeota archaeon	acetyl-CoA synthetase (ADP forming)	RLE53850.1	Archaea/Verstraetearchaeota/undefined
Thermoprotei archaeon	acetyl-CoA synthetase (ADP forming)	RLE57312.1	Archaea/Thermoproteota/Thermoprotei
Hyperthermus butylicus	CoA_binding protein	WP_011822483.1	Archaea/Thermoproteota/Thermoprotei
Staphylothermus marinus	CoA_binding protein	WP 011838751.1	Archaea/Thermoproteota/Thermoprotei
Candidatus Korarchaeum cryptofilum	CoA_binding protein	WP_012308855.1	Archaea/Korarchaeota/Korarchaei
Fervidicoccus fontis	CoA_binding protein	WP_014557871.1	Archaea/Thermoproteota/Thermoprotei
Thermococcus cleftensis	acetate-CoA ligase	WP_014789415.1	Archaea/Thermoproteota/Thermoprotei
Staphylothermus hellenicus	CoA_binding protein	WP_052833590.1	Archaea/Thermoproteota/Thermoprotei
Thermofilum adornatum	CoA_binding protein	WP_052886942.1	Archaea/Thermoproteota/Thermoprotei
Thermococcus celericrescens	acetate-CoA ligase	WP_058938949.1	Archaea/Thermoproteota/Thermoprotei
Thermosphaera aggregans	CoA_binding protein	WP_193435632.1	Archaea/Thermoproteota/Thermoprotei
Thermogladius calderae	CoA_binding protein	WP_202945702.1	Archaea/Thermoproteota/Thermoprotei
Caldisphaera lagunensis	acetyltransferase	WP_015232801.1	Archaea/Thermoproteota/Thermoprotei
Metallosphaera sedula	acetyl-CoA synthetase (ADP forming) alpha domain containing protein	WP_240252923.1	Archaea/Thermoproteota/Thermoprotei
Sulfolobus islandicus	acetyl-CoA synthetase (ADP forming) alpha domain containing protein	WP_012715981.1	Archaea/Thermoproteota/Thermoprotei
Sulfolobus solfataricus	acetyl-CoA synthetase (ADP forming) alpha domain containing protein	WP_009992015.1	Archaea/Thermoproteota/Thermoprotei

Infirmifilum uzonense	acetyltransferase	WP_191118496.1	Archaea/Thermoproteota/Thermoprotei
